# Discriminative Prediction of A-To-I RNA Editing Events from DNA Sequence

**DOI:** 10.1371/journal.pone.0164962

**Published:** 2016-10-20

**Authors:** Jiangming Sun, Yang De Marinis, Peter Osmark, Pratibha Singh, Annika Bagge, Bérengère Valtat, Petter Vikman, Peter Spégel, Hindrik Mulder

**Affiliations:** 1 Department of Clinical Sciences in Malmö, Unit of Molecular Metabolism, Lund University Diabetes Centre, CRC, Malmö, Sweden; 2 Department of Clinical Sciences in Malmö, Unit of Diabetes and Endocrinology, Lund University Diabetes Centre, CRC, Malmö, Sweden; 3 Centre for Analysis and Synthesis, Department of Chemistry, Lund University, Lund, Sweden; Tianjin University, CHINA

## Abstract

RNA editing is a post-transcriptional alteration of RNA sequences that, *via* insertions, deletions or base substitutions, can affect protein structure as well as RNA and protein expression. Recently, it has been suggested that RNA editing may be more frequent than previously thought. A great impediment, however, to a deeper understanding of this process is the paramount sequencing effort that needs to be undertaken to identify RNA editing events. Here, we describe an *in silico* approach, based on machine learning, that ameliorates this problem. Using 41 nucleotide long DNA sequences, we show that novel A-to-I RNA editing events can be predicted from known A-to-I RNA editing events intra- and interspecies. The validity of the proposed method was verified in an independent experimental dataset. Using our approach, 203 202 putative A-to-I RNA editing events were predicted in the whole human genome. Out of these, 9% were previously reported. The remaining sites require further validation, e.g., by targeted deep sequencing. In conclusion, the approach described here is a useful tool to identify potential A-to-I RNA editing events without the requirement of extensive RNA sequencing.

## Introduction

RNA editing is a post-transcriptional processing of the RNA molecule, involving insertions and deletions of bases, as well as base substitutions. RNA editing has been observed in eukaryotes as well as viruses, archaea and prokaryotes, and has attracted broad interest recently [1–4]. Until now, two types of RNA editing have been described in mammals. The first is C-to-U editing catalyzed by the apolipoprotein B mRNA editing enzyme, catalytic polypeptide-like (APOBEC) family of enzymes; its frequency is quite low. The second is A-to-I editing, where adenosine is deaminated to inosine by the adenosine deaminases acting on RNA (ADAR) family of enzymes; this is the predominating type of RNA editing. In 2011, widespread differences between human RNA and DNA sequences were found [5]. From this finding, the existence of a novel form of RNA editing was inferred, providing a yet unexplored dimension of genomic variation. However, the existence of non-canonical RNA editing in humans is still debated [6, 7]. RNA editing, particularly A-to-I events, is widespread in humans. These events have been suggested to be linked to miRNA-mediated post-transcriptional regulation, although removal or formation of miRNA recognition sites, and/or alterations in miRNA sequences also seem to be frequent [8, 9].

Accurate identification of RNA editing events from high throughput sequencing data still remains a great challenge. Current comparative methods usually map short reads to a reference genome or transcriptome, followed by removal of identical reads, filtering of low quality reads, calling variants and discarding of known single nucleotide polymorphisms (SNPs) [7, 10–14]. Mapping massive numbers of short reads to a reference genome is time-consuming and only a few pipelines and computational tools are publicly available for RNA editing calling [15]. Moreover, it is difficult to discriminate RNA editing events from novel SNPs since DNA sequencing is not routinely performed in conjunction with RNA sequencing. In fact, a substantial number of RNA editing events has been annotated as SNPs in dbSNP [16]. Prediction of C-U editing has been carried out in plant organelles [17] and in physarum mitochondria [18], using homologous information of protein sequences. However, most of these methods are not capable of predicting RNA editing events that result in synonymous substitutions and editing in non-coding regions.

**Fig 1:**
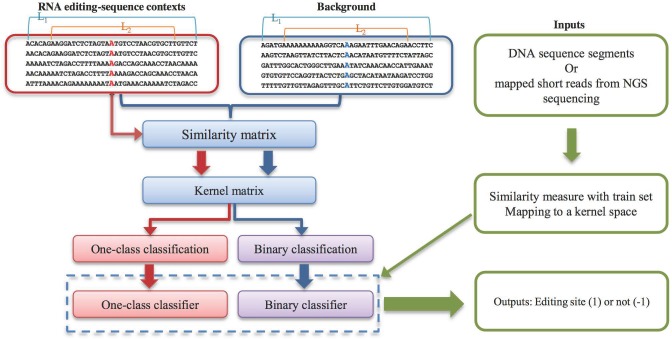
The flowchart of the proposed method. First, we obtain flanking sequences surrounding the known editing sites and background sets from the reference genome. Second, we measure the similarity of sequence segments pairs (background sets are not used during the training process for one-class classification). Then the generated similarity matrix is mapped to string kernel space. For this kernel matrix, the training process tries to find a maximal margin hyperplane, which is usually achieved by quadratic programming. Based on a generated model from the training process, prediction can be made, using any given sequence or mapped short reads from next generation sequencing by extracting adenine centered segments. L_1_: length used for calculating edit distance. L_2_: length used for calculating Hamming distance.

To overcome some of these problems, we here introduce a machine learning approach (Fig 1) to detect A-to-I RNA editing events from a genome sequence alone. Hence, this approach does not require RNA sequencing.

## Methods and Materials

### Data

The Darned database (Sep 2013), used in this study, was created by Kiran and Baranov [19] [20]. It includes data generated by a variety of methods: (1) bioinformatics analysis of discrepancies between cDNA sequences and genomic sequences; (2) analysis of SNP data; (3) analysis of miRNA; and (4) results of high-throughput sequencing of RNA and DNA samples from the same tissues [20]. After that, sites of RNA editing events were mapped to a reference human genome. The reliability of those methods was, however, not provided in the Darned database. The database collects RNA editing events from drosophila melanogaster (fruit fly), mouse and human. We extracted reference genome sequences for the coordinate of known A-to-I RNA editing events, including flanking sequences with a length of 5nt (nucleotide), 10nt, 15nt, 20nt and 25nt, as positive data set. The negative data set was created from sequences of the same length that were randomly generated by 1 nucleotide per 10000 nucleotides from the whole reference genome. This frequency is close to the number of current known human A-to-I RNA editing events divided by size of human-sequenced base pairs: ~1.2e-4. Among them we kept adenine-centered sequences and filtered instances included in the positive set. For mouse, one-fifth instances were reselected from the randomly generated data sets as negative data set for training. Similarly, negative human data set were reselected to match the entries of the positive human data set. The number of sequences in the positive and negative data sets was for fruit fly 1959 (S1 Table) and 4535, respectively. For mouse, there were 8059 (S2 Table) sequences in the positive data sets and 15475 sequences in the negative data sets. For human, there were 26228 (S3 Table) and 26228 sequences in the positive and the negative data set, respectively. Binary classifications employed all these three datasets as training sets but only positive data sets were used for training of one-class classification.

### Clustering

CD-HIT [21] (version 4.6) was used to cluster editing-sequence contexts. Within each species, command CD-HIT-EST compares DNA sequence segments with default settings and a threshold of 0.8. This threshold means that one sequence is redundant if identity is greater than 0.8 with another. Command CD-HIT-EST-2D with default settings and a cut off of 0.8 is used to compare editing-sequence contexts between two species. It identifies DNA sequence segments in one species that are similar to another species when identity is above 0.8. In both commands, strand specificity is not considered, since both +/+ and +/- alignments are performed.

### String distances

The edit distance, also called Levenshtein distance [22], counts how many insertions, deletions and substitutions that are required to yield identical sequences. Unlike the edit distance, the Hamming distance [23] only allows substitutions. In this study, we used a combination of edit- and Hamming distances to measure the similarity between two DNA sequence segments (Eq 1):

$$D=w\times \frac{1}{L_{1}}\times D_{Edit}+(1-w)\times \frac{1}{L_{2}}\times D_{Hamming}$$ 1

where D_Edit_ is the edit distance for the L_1_-length and D_Hamming_ is the Hamming distance for the L_2_-length pair sequence segments. L_1_ and L_2_ are lengths used for calculating edit distance and Hamming distance, respectively. L_1_ can be greater, equal or less than L_2_. The w is the weight varying from 0 to 1. When w = 0, only the Hamming distance contributes to the combinational distance, D. On the contrary, when w = 1, only the edit distance contributes to D. The higher the value of D is, the lower the similarity between the sequences is.

### String kernel

String kernel is a kernel function that operates on strings type data. We use a string kernel function in LIBSVM [24] to convert string data into a vector space. A kernel function K(a,b) can be written as (Eq 2):

$$K(a,b)=exp(-gamma\times {D(a,b)}^{2})$$ 2

where D is the combinational distance derived from Eq 1. The gamma parameter defines how far the influence of a single training instance reaches, with low values meaning”far” and high values meaning "close”. We used a default gamma value of 0.1 as LIBSVM did in this study.

### One-class classification

One-class SVM was used to build a classifier that recognizes A-to-I RNA editing event. In contrast to traditional SVMs, one-class SVMs attempt to learn a decision boundary that achieves the maximum separation between the points and the origin [25]. According to Schölkopf et al. [25], this results in binary values of 1 as editing event, and -1 as non-editing event in this study. In one-class SVM, an important parameter nu (varying from 0 to 1) was an upper bound on the fraction of training errors and a lower bound of the fraction of support vectors [26]. A small value of nu (e.g. nu = 0.1) indicates a small training error and few outliers in the train data, compared to a greater value of nu (e.g. nu = 0.5).

### Evaluation metrics

N-fold cross validation was used to evaluate the model. Six common criteria, sensitivity (Sn), specificity (Sp), accuracy (Acc), positive predictive value (PPV), Matthew's Correlation Coefficient (MCC) and geometric mean (G mean) [27], were used to evaluate the performance of the binary classification. The MCC and G mean metrics consider the performance on both the positive and the negative data sets. High MCC or G mean values indicate high accuracies on both positive and negative data sets, making these criteria appropriate to learn on imbalanced data. Sn, Sp, Acc, PPV, MCC and G mean are defined as (Eqs 3–8):

Sn=TP/P 3

Sp=TN/N 4

Acc=(TP+TN)/(P+N) 5

PPV=TP/(TP+FP) 6

$$MCC=\left(TP\times TN-FP\times FN\right)/\sqrt{P\times N\times (TP+FP)(TN+FN)}$$ 7

$$G\;mean=\sqrt{Sn\times Sp}$$ 8

Where TP is the number of true positive, FP is the number of false positive, TN is the number of true negative, FN is the number of false negative, P is the total number of positive instances and N is the total number of negative instances.

### A-to-I RNA editing events calling and validation

A-to-I RNA editing events were called from RNA sequencing (RNA-seq) data generated from human islets from 184 donors (Data partly published and deposited in the Gene Expression Omnibus (GEO) database, accession no. GSE50244). Details of RNA-seq and exome sequencing (Exome-seq) experiments can be found in a recent study [28]. 101-bp long paired-end reads were aligned to hg19, using STAR [29]. GATK [30] was employed for reads realigned, quality base scores recalibrated and variants calling. An editing event should have a Phred quality score (Q) greater than 30, more than 8 reads per base in at least two sample and an editing degree greater than 5%. Considering that reference and alternative alleles were always given on the forward strand by GATK, T-to-C events were treated as A-to-I events on the opposite strand. Using the same settings, Exome-seq data derived from islets from 12 human donors (Data unpublished) with corresponding RNA-seq data (Data unpublished) was used to validate A-to-I RNA editing events.

### Software availability

The source for one-class and binary classifications is modified based on LIBSVM. It is available at http://figshare.com/projects/Discriminative_prediction_of_A-to-I_RNA_editing_events_from_DNA_sequence/15510/. Training and testing datasets are also deposited within this link.

## Results

### Data description

**Fig 2:**
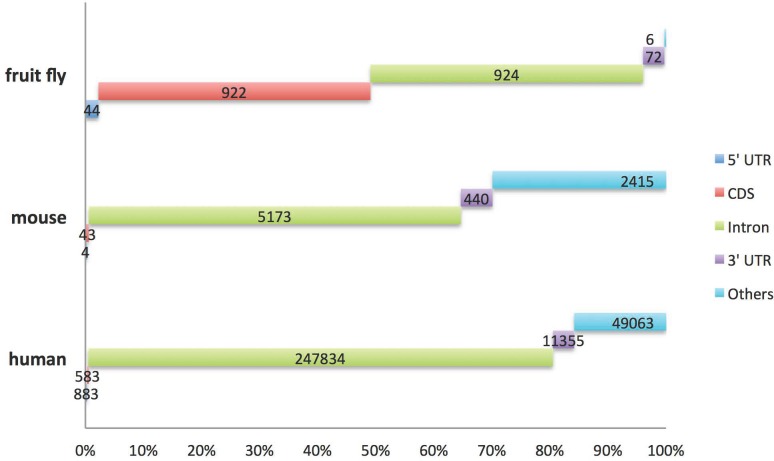
The genomic distribution of A-to-I RNA editing events. Gene annotation here was derived from the DARNED (http://darned.ucc.ie/help/). 5’ UTR: 5’ untranslated regions. 3’ UTR: 3’ untranslated regions. CDS: coding region of a gene. An "other" region refers to an RNA editing coordinate not part of an intron or exon.

First, we made a descriptive analysis using the Darned database [19] (Fig 2). This analysis revealed that A-to-I RNA editing events were most frequent in introns, followed by regions that were neither introns nor exons (others) or 3’ untranslated regions (3’ UTR) in both mice and humans. A substantial number of editing events in introns and in 3’ UTR (440 RNA editing events in mouse and 11355 in human) implied that RNA editing may play an important role in regulation of miRNA and splicing. In contrast, the genome distribution of A-to-I RNA editing events in fruit fly was radically different; roughly half of the editing events occurred in introns while the other half occurred in coding regions (coding DNA sequences; CDS). Very few A-to-I RNA editing events were found in CDS in mouse and human.

Subsequently, 10 nucleotides (nt) long flanking sequences surrounding the coordinate of the A-to-I RNA editing event (editing-sequence contexts) were extracted from the reference genome (fruit fly: dm3, mouse: mm10, human: hg19). A comparison of similarity among the three species by CD-HIT [21], a program for clustering large sets of nucleotide sequence (see Methods and Materials) (Table 1), revealed little similarity between the editing-sequence contexts in fruit fly and mouse (1.3% of known A-to-I editing events in fruit fly and 0.4% of known A-to-I editing events in mouse; sequence identity cut off = 0.8). Conversely, editing-sequence contexts in mouse and human were more similar (31.9% of known A-to-I editing events in mouse and 30.6% of known A-to-I editing events in humans were similar). Only one editing-sequence context, located on the X chromosome in mouse and humans and on the 2R chromosome in fruit fly, was similar among all the three species.

**Table 1:** Similarity of editing-sequence contexts in fruit fly, mouse and human. Each row shows the number of A-to-I editing-sequence contexts in species similar with those in fruit fly, mouse and human. Values in brackets refer to training datasets in this study.

	Fruit fly	Mouse	Human
Records in fruit fly	1959 (1690)	26 (12)	233 (102)
Records in mouse	30 (13)	8059 (4187)	2574 (673)
Records in human	931 (115)	94366 (915)	308272 (26228)

Next, we investigated the redundancy of A-to-I editing-sequence contexts in each species by using CD-HIT with a cut off of 0.8 (see Methods and Materials). For the fruit fly, mouse and humans, 1 690 out of 1 959 instances, 4 187 out of 8 059 instances and 26 228 out of 308 272 instances, respectively, remained.

### Parameter selection and the performance of prediction on fruit fly, mouse and human genome

**Fig 3:**
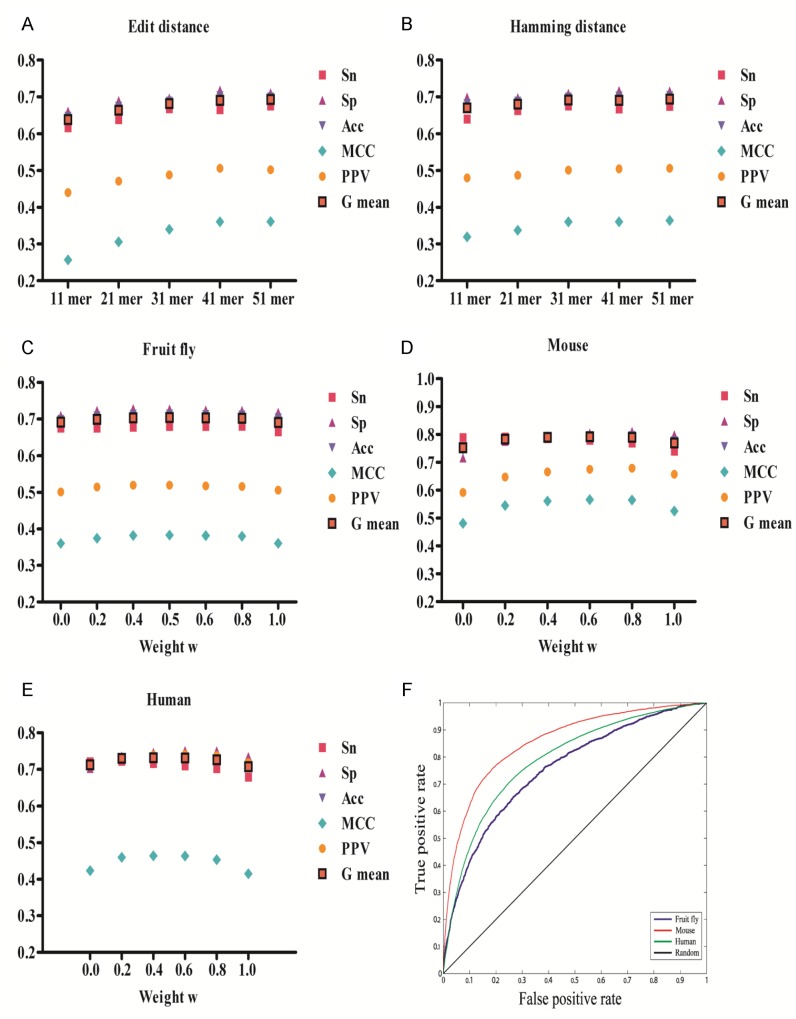
The performance of the five-fold cross validation. Performances on fruit fly with 11-mer, 21-mer, 31-mer, 41-mer and 51-mer length of editing-sequence contexts using (A) edit distance and (B) Hamming distance. The longer the flanking sequences were, the higher the specificity of editing-sequence context was. Based on the performance of the prediction on fruit fly data, 10 flanking sequences could provide reasonable enough information for prediction while 20 flanking nucleotides improved the prediction dramatically. Performances on (C) fruit fly, (D) mouse and (E) human when the parameter w is varied from 0 to 1. Based on evaluation of Sn and G mean, the best weight for fruit fly, mouse and human is 0.5, 0.2 and 0.2, respectively. (F) ROC curve for fruit fly (blue line), mouse (red line) and human (green line) with five-fold cross validation. The gray solid line shows the random guessing results. The greater the area under ROC curve, the better the performance of prediction.

To find the optimal length of editing-sequence contexts for prediction, we employed the fruit fly as model. We extracted 5nt, 10nt, 15nt, 20nt and 25nt long flanking sequences surrounding the A-to-I RNA editing events (11mer, 21mer, 31mer, 41mer and 51mer editing-sequence contexts) from the fruit fly genome (dm3). Considering that positive instances and negative instances in the fruit fly data were imbalanced, we oversampled the positive data set 2.31-fold. As displayed in Fig 3A and 3B, we observed that longer sequence segments improved the performance of both edit and Hamming distances (S4 Table). For the edit distance, the performance of prediction, using 5-fold cross-validation (Sn = 0.665, Sp = 0.719, Acc = 0.703, MCC = 0.360, PPV = 0.506 and G mean = 0.691), was close to a stationary point when predicting on 41mer editing-sequence contexts. For the Hamming distance, the sensitivity (Sn = 0.675) reached maximum and the other measures of performance (Sp = 0.710, Acc = 0.699, MCC = 0.360, PPV = 0.501 and G mean = 0.692) were close to a stationary point, when prediction was based on 31mer editing-sequence contexts. Consequently, 41mer editing-sequence contexts (the Hamming distance was measured on 31mer inner sequence contexts) were chosen to study the prediction of A-to-I RNA editing events in all three species. With this setting, the prediction (w = 0.5) for fruit fly yielded an Sn of 0.680, Sp of 0.728, Acc of 0.714, MCC of 0.383 and a G mean of 0.704.

A 5-fold cross validation was performed to evaluate which weight w to use for fruit fly, mouse and human data. An oversampling approach was used to balance positive and negative instances for mouse (1.92-fold oversampling for the positive data set). The positive human data set was assembled from the representative sequences of clusters (see Data description). The majority of performance criteria reached maximum when w equaled 0.5 for fruit fly data (Fig 3C). For mouse and human data (Fig 3D and 3E), the Sn initially increased but then gradually declined. On the other hand, when w was varied from 0 to 0.8, the Sp improved dramatically, by 13.3% for mouse and 7.1% for human. The Sn reached a maximum when w = 0.2 (Sn = 0.791 and 0.722 for mouse and human, respectively). The other performance criteria were close to a stationary point (Acc = 0.781 and 0.730, MCC = 0.545 and 0.460, PPV = 0.647 and 0.733, G mean = 0.783 and 0.730, for mouse and human data, respectively). Consequently, w was set to 0.5 for fruit fly and 0.2 for both mouse and human, to allow for correct recognition of most RNA editing events.

A receiver operating characteristic (ROC) curve was plotted for fruit fly (w = 0.5), mouse (w = 0.2) and human (w = 0.2). The AUC (area under the curve) values for fruit fly, mouse and human were 0.756, 0.857 and 0.791, respectively. In line with results displayed in Fig 3C–3E, the prediction based on mouse and human data reached a lower false positive rate and higher true positive rate than predictions based on fruit fly data (Fig 3F).

Using results of five-fold cross-validation for drawing ROC curve when weight w = 0.2, we evaluated the performance of the binary classifier separately on regions of Alu elements and non-Alu elements of human A-to-I RNA editing events, respectively. 6544 out of 7375 events (88.7%) in Alu regions and 12500 out of 18853 events (66.3%) in non-Alu regions were correctly recognized, respectively (S5 Table).

### Predicting A-to-I RNA editing events using positive data only

Not surprisingly, one-class classification of the positive data sets, using a default nu value (see Methods and Materials) of 0.5, was less accurate (Acc value of 0.489, 0.495 and 0.498 for fruit fly, mouse and human, respectively) than binary classification. This is likely due to the absence of a negative control. Therefore, the performance of the model was studied on the positive data set, using 5-fold cross-validation and varying values of nu from 0.1 to 0.5. Predictions on the negative data sets were used to test the reliability of the model. The accuracy decreased roughly 2-fold for all species in positive data sets (0.871 to 0.489 for fruit fly, 0.889 to 0.495 for mouse and 0.897 to 0.498 for human) while it increased roughly 3-fold in negative data sets (0.262 to 0.737 for fruit fly, 0.358 to 0.861 for mouse and 0.154 to 0.660 for human) when nu was varied from 0.1 to 0.5. For all cases, the prediction on negative data sets from mouse was about 10% higher than that from fruit fly (Table 2). However, the performances of prediction on negative data sets were poor when nu was small. At this point, it is necessary to find a suitable value of nu to obtain a favorable balance of false/true positive and negative rates. Typically, a small nu value will cluster most A-to-I RNA editing events in the same class. This is probably associated with high rates of both true and false positives. A higher nu is likely to treat a larger number of A-to-I RNA editing events as outliers since they are distant from the center of the cluster.

**Table 2:** The performances of one-class classification.

Species	*nu*	Positive data set	Negative data set	Human positive data set	Human negative data set	Independent data set
Fruit fly	0.1	0.871	0.262	0.803	0.290	0.776
	0.2	0.769	0.416	0.652	0.459	0.621
	0.3	0.674	0.540	0.538	0.590	0.483
	0.4	0.577	0.651	0.429	0.695	0.431
	0.5	0.489	0.737	0.335	0.781	0.397
Mouse	0.1	0.889	0.358	0.732	0.367	0.724
	0.2	0.791	0.566	0.574	0.578	0.569
	0.3	0.693	0.695	0.449	0.719	0.379
	0.4	0.593	0.786	0.347	0.815	0.293
	0.5	0.495	0.861	0.261	0.881	0.207
Human	0.1	0.897	0.154	-	-	0.879
	0.2	0.798	0.296	-	-	0.828
	0.3	0.697	0.421	-	-	0.793
	0.4	0.599	0.545	-	-	0.724
	0.5	0.498	0.660	-	-	0.466

Independent data coming from the Supplementary S8 Table (Peng et al. 2012) by filtering out non- A-G editing and non-A reference alleles (See Results)

### Human A-to-I RNA editing events can be predicted from animal models

Next, we employed one-class classification models built on fruit fly and mouse data to study A-to-I RNA editing events in humans (Table 2). With a small nu, the model trained on fruit fly data was more efficient in correctly predicting A-to-I RNA editing events in human data than the corresponding model trained on mouse data. However, the model derived from fruit fly data also incorrectly assigned a large number of negative instances as editing events. As shown in Table 3, the binary classification gave reasonable results for all species, correctly recognizing 80% of the negative instances. The model trained on mouse data outperformed the model trained on fruit fly data (2.1%, 6.8% and 4.7% improvement in Sn, MCC, and PPV, respectively) in predicting A-to-I RNA editing events in the human data.

**Table 3:** Predicting RNA editing events in human using animal models.

Model	Sn	Sp	Acc	MCC	PPV	G mean
Fruit fly	0.574	0.762	0.668	0.342	0.707	0.661
Mouse	0.595	0.806	0.700	0.410	0.754	0.692
Fruit fly + Mouse	0.622	0.784	0.703	0.412	0.743	0.699

Due to the differences between models trained on the different species, we trained a model on combined fruit fly and mouse data. With this mixed-model, prediction of editing events in human data was improved with respect to sensitivity (yielding an Sn of 0.622, MCC of 0.412 and G mean of 0.699). Remarkably, the PPV values were above 0.700 in all three animal models, indicating a low false discovery rate. Hence, models generated on fruit fly and mouse data reliably recognized human editing events.

### Prediction and comparison on validated human A-to-I RNA editing events

To assess the predictive capability of our models, we culled human A-to-I editing events from a Sanger sequencing data set (57 out of 58 editing events were validated) [9]. Out of such 58 A-to-I RNA editing events, 46 (79.3%) were correctly recognized by a model that was trained on human data. The accuracy of the prediction, using models trained on fruit fly, mouse and combined fruit fly and mouse data, was 0.724, 0.690 and 0.724, respectively (S6 Table). One candidate RNA editing event (chromosome: 1, coordinate: 28534767, strand: +), identified by computational pipelines treating sequencing data, was recognized as a false positive when validated by Sanger sequencing. In contrast, our approach correctly recognized it, using models trained on data from both fruit fly and mouse.

One-class classification (Table 2) recognized a larger number of these validated RNA editing sites than binary classification (77.6%, 72.4% and 87.9% for models based on fruit fly, mouse and human data, respectively, with nu = 0.1). However, one-class classification trained with a small nu value (for example, nu = 0.1 for fruit fly) may generate a large number of false positives, which clearly limits its application. This notwithstanding, one-class classification could still be useful as a first step to filter out most unlikely editing sites.

Our approach outperformed an existing predictor InosinePredict [31], whose accuracies were 72.4% and 60.3%, respectively based on preferences for human ADAR1 (hADAR1) and human ADAR2 (hADAR2). An accuracy of 77.6% could be obtained using InosinePredict if we combined predicted results from both hADAR1 and hADAR2 (Table 4).

**Table 4:** Comparison of predictors.

Model	Accuracy
Our approach_Binary_classification_	0.793
Our approach_One-class_classification_	0.879
InosinePredict_hADAR1_	0.724
InosinePredict_hADAR2_	0.603
InosinePredict_hADAR1 + hADAR2_	0.776

InosinePredict: cutoff of unedited (A) sites: 9.6% for hADAR1 (human ADAR1), and 21% for hADAR2 (human ADAR2). The InosinePredict_hADAR1 + hADAR2_ combined right predictions either based on the model of hADAR1 or hADAR2.

### Identification of putative editing sites in the whole human genome

**Fig 4:**
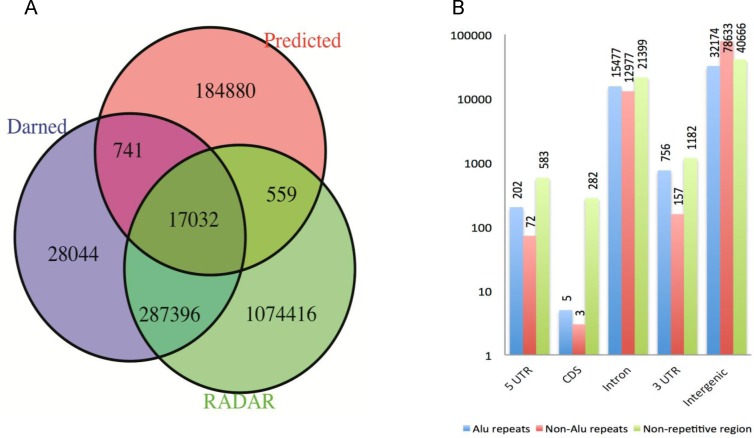
The putative A-to-I RNA editing events. (A) Overlap between the predicted RNA editing events (red) with Darned (blue) and RADAR (green). Notice that RADAR only collects A-to-I RNA editing events. (B) The genomic distribution of predicted RNA editing events. The genomic regions are annotated by UCSC known genes and repeat masker (hg19, http://genome.ucsc.edu/). One genome coordinate position may have multi- annotations. 5’ UTR: 5’ untranslated regions. 3’ UTR: 3’ untranslated regions. CDS: coding region of a gene. Intergenic: intergenic region. Alu repeat: a short stretch of DNA originally characterized by the action of the Arthrobacter luteus restriction endonuclease.

First, we took 26 228 representative editing-sequence contexts from clusters obtained by CD-Hit (see Data description) as seeds. This generated 438 339 exactly matched segments from the whole human genome (hg19). Second, we used one-class support vector machine (SVM) (nu = 0.1) to remove most unlikely editing sites. Third, we applied binary classification, trained on human data, to identify the most likely editing events on sequence contexts that passed one-class classification. Thereby, 203 212 editing sites were identified by prediction. Among these, only 2 715 were found in dbSNP (Build 139), 17 773 were found in the Darned database and 17 591 were found in the RADAR database [32] while 17 032 were found in both databases, which showed a low overlap between editing sites in previous studies (less than 10%). Thus, 91.0% of the potential editing sites identified by our approach were novel (Fig 4A).

The genomic distribution of these putative RNA editing sites is shown in Fig 4B. These editing sites were rarely found in exons (1%), but quite frequently in intergenic region (74.5%) and introns (24.5%). The number of editing sites in 5’ UTR, CDS and 3’ UTR were 860, 290 and 2104, respectively. 174 out of 290 sites in CDS were non-synonymous substitutions (S7 Table). We found 33 editing sites in pri-miRNA; within them, 24 sites were maintained in mature miRNA, as assessed by the miRBase [33]. None of the predicted sites were found in the genomic positions of small nuclear RNA (snRNA) and small nucleolar RNAs (snoRNAs). Interestingly, we found 6 972 A-to-I RNA editing events in long intergenic non-coding RNAs (lincRNA). Out of these, 6 075 were novel, and not found in the Darned database. Ten sites were found in tRNA based on a genomic tRNA database GtRNAdb [34]. Considering that tRNA editing is different from editing events on double-stranded RNA, we removed these tRNA editing events from the putative set. Thus, 203 202 editing sites were identified by our approach (S1 Dataset).

### Validation of exonic A-to-I RNA editing events in human pancreatic islets

**Fig 5:**
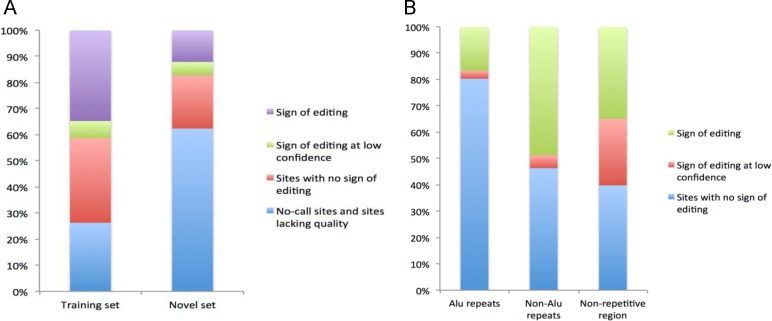
Validation of putative A-to-I RNA editing events. (A) A-to-I RNA editing events observed in human islets for 3893 known sites (training set) and 561 putative sites (novel set). In the training set, 1352 sites show signs of editing, 257 sites show signs of editing but at a low confidence, 1260 sites including 506 SNPs do not show any sign of editing and 1024 no-call sites. In the novel set, 68 sites show signs of editing, 29 sites show signs of editing but at a low confidence, 114 sites including 8 SNPs do not show any sign of editing and 350 no-call sites. (B) The observed A-to-I RNA editing events in Alu repeats, non-Alu repeats and non-repetitive region in human islets. There are 11, 2 and 53 sites in Alu repeats, 20, 2 and 19 sites in Non-Alu repeats and 36, 26 and 41 sites in non-repetitive region show signs of editing, signs of editing at low confidence and no sign of editing, respectively.

There were 561 putative A-to-I RNA editing events in exons out of the reported putative events left after removing overlap with DARNED and RADAR. First, we validated these sites using RNA sequencing of human islets from 184 donors (Fig 5). 322 were sites of low quality (Q<30) mapped reads, 1 site with only one allele count and 28 were no-call sites. These sites were not considered for further analysis. Finally, 210 sites were kept for validation. Out of these, 103, 41 and 66 sites were located in non-repetitive regions, non-Alu repeats and Alu repeats, respectively. 36 showed signs of editing in non-repetitive regions in human islets. In addition, 28 events were observed with a low confidence (<8 reads per base or observed in only one individual), and 8 events were also sites of common SNPs. 20 events showed signs of editing in non-Alu repeats. Only 11 sites showed signs of editing in Alu repeats (S8 Table).

In contrast, we also investigated all (3893) known exonic A-to-I RNA editing events in the human training set. After filtering 902 low quality sites, 122 no-call sites and 506 SNPs, 1352 out of 2363 (57.2%) known editing sites were identified by RNA-seq in the human islets alone (Fig 5A and S9 Table). Of these, seven editing events were also reported in a previous study [28], which was based on data from islets from 82 human donors; our extended study sample of 184 donors included these 82 donors.

After this initial validation using RNA-seq alone, we further validated these novel exonic A-to-I RNA editing events, using novel Exome-seq and RNA-seq data from 12 recently analyzed human islet donors. We identifed 47 editing sites by RNA-seq, out of which 35 could be validated comparing genotypes derived from Exome-seq with that of RNA-seq. However, genotypes of 11 sites could not be inferred from Exome-seq, and were thus potential artefacts from sex chromosomes. Only one site failed to be validated. Interestingly, 4 SNPs were also found to be sites of A-to-I RNA editing events in human islets (Table 5).

**Table 5:** Validation of putative A-to-I RNA editing events in 12 human islets using RNA-seq and Exome-seq.

Chrom	Position	dbSNP ID	Ref	Alt	Alu repeats	Non-Alu repeats	Non-repetitive	Signs of editing using RNA-seq alone	Signs of editing using Exome-seq and RNA-seq
1	16996		T	C	No	No	Yes	Yes	Yes
1	136145		T	C	No	No	Yes	Yes	Yes
1	136176		T	C	No	No	Yes	Yes	Yes
1	327178		A	G	No	No	Yes	Yes	Yes
1	327209		A	G	No	No	Yes	Yes	Yes
1	146033351		T	C	No	Yes	No	Yes	Yes
1	146033357		T	C	No	Yes	No	Low confidence	Yes
1	146466935		A	G	No	Yes	No	Low confidence	Yes
1	148003734		T	C	No	Yes	No	Yes	Yes
1	148251061		T	C	No	Yes	No	Yes	Yes
1	148251067		T	C	No	Yes	No	Yes	Yes
1	148757492		A	G	No	Yes	No	Low confidence	Yes
1	149671816		A	G	No	No	Yes	Low confidence	Yes
2	114352638	rs377338570	A	G	No	No	Yes	Yes	Yes
3	188598721		A	G	Yes	No	No	Yes	Lack of coverage
3	197940920		A	G	No	No	Yes	Low confidence	Yes
4	3511496	rs10020994	T	C	No	No	Yes	Yes	Lack of coverage
5	43527381	rs71629179	T	C	No	No	Yes	Yes	Failed
5	69744694		A	G	Yes	No	No	Low confidence	Lack of coverage
5	180753793		A	G	No	No	Yes	Low confidence	Yes
5	180753824		A	G	No	No	Yes	Low confidence	Yes
6	36109404		A	G	Yes	No	No	Yes	Lack of coverage
7	76681891		A	G	No	No	Yes	Low confidence	Yes
7	102135806	rs377051321	A	G	No	No	Yes	Yes	Yes
7	102234975	rs372070045	A	G	No	No	Yes	Yes	Yes
7	128293801		A	G	No	No	Yes	Yes	Yes
8	42876550		A	G	No	No	Yes	Yes	Yes
9	18481	rs11555811	T	C	No	No	Yes	Yes	Yes
10	47228601		T	C	Yes	No	No	Low confidence	Lack of coverage
11	128359		T	C	No	No	Yes	Low confidence	Yes
11	128390		T	C	No	No	Yes	Low confidence	Yes
12	87267		A	G	No	No	Yes	Yes	Yes
12	88651		A	G	No	No	Yes	Yes	Yes
15	102514167		A	G	No	No	Yes	Yes	Yes
16	66679		T	C	No	No	Yes	Yes	Yes
16	21443512		T	C	Yes	No	No	Low confidence	Lack of coverage
16	21875788		T	C	Yes	No	No	Low confidence	Lack of coverage
16	90237172		A	G	No	No	Yes	Yes	Yes
16	90237203		A	G	No	No	Yes	Low confidence	Yes
17	44412592		T	C	No	No	Yes	Yes	Yes
18	60995796		T	C	Yes	No	No	Yes	Yes
19	198277		T	C	No	No	Yes	Low confidence	Yes
19	198308		T	C	No	No	Yes	Low confidence	Yes
X	15802285		A	G	No	No	Yes	Yes	Lack of coverage
X	155251357		A	G	No	No	Yes	Yes	Lack of coverage
X	155252758		A	G	No	No	Yes	Yes	Lack of coverage
Y	59354363		A	G	No	No	Yes	Yes	Lack of coverage

Chrom: Chromosome. Ref: reference allele. Alt: alternative allele. All alleles refer to forward strand. Low confidence means that reads per base are smaller than 8.

## Discussion

Identification of RNA editing events is of great importance for the understanding of post-transcriptional regulation. Currently, most editing sites have been identified by large scale sequencing efforts. However, these studies are expensive and time-consuming. In addition, it is difficult to distinguish editing events from SNPs and sequencing errors. Furthermore, whereas we today have access to huge amounts of genomic data, we rarely have access to both DNA and RNA sequences from the same individual. Currently, a targeted analysis of RNA editing remains a challenge, since information on where they occur is lacking. A recent study of ultra-deep Alu-targeted sequencing showed that the scope of editing is much greater than expected [35]. Most identified sites exhibited a low level (<1%) of editing. It was not possible to provide a comprehensive overview of the whole human genome. In an attempt to resolve this problem, we here introduce a machine learning approach to predict A-to-I RNA editing events from genomic data. It can be used to identify putative editing sites or to test whether RNA editing is likely to occur in a given DNA sequence. With this approach, the accuracy of prediction was generally determined by the source and quality of training data. The more representative the real distribution of training data was, the more powerful the prediction was. To this end, we used known editing events in fruit fly, mouse and human as training sets. These data were diverse within the species itself but conserved between species. This was supported by: 1, a substantial number of clusters following clustering of editing-sequence contexts for fruit fly, mouse and human; 2, mouse and human shared a substantial number of similar editing-sequence contexts; 3, mouse and human displayed a similar genomic distribution of RNA editing events; 4, about 60% of human editing events could be predicted by training on data from fruit fly, mouse or a mix of them. The great similarity between mouse and human suggested that the mouse could be used as a model to study RNA editing in the human.

To avoid overfitting, we performed predictions based on models derived from one species on data from other species in addition to validation, using Sanger sequencing-verified RNA editing events. To this end, models trained on human data or combined data from fruit fly and mouse correctly recognized more than 70% of verified RNA editing sites. The successful identification on this independent data set validated the proposed method. From the study on currently known RNA editing events, we concluded that A-to-I RNA editing events could be predicted by use of the DNA sequence. However, we also believe that use of more data is likely to generate a higher accuracy of prediction.

Considering that the negative data sets that we used for binary classification may contain novel editing events, we used a one-class classification approach to overcome this problem. With this approach, we could predict A-to-I RNA editing events using only a positive data set. However, the accuracy of this approach was slightly lower than for binary classification. This was likely due to that most editing events were merged into a single cluster. The non-editing events might be mistaken as editing events if they were located in the distribution of this one cluster. One solution to this problem would be to divide A-to-I RNA editing events into numbers of clusters and to perform one-class classification on each cluster. Another possible solution to address false positive is enlarging negative data sets used in the binary classification. However, this approach would result in extremely low sensitivity and MCC.

String distances have for long been applied to measure similarity of DNA or protein sequences [36]. We found that the length of the flanking sequence was an important parameter to obtain a high specificity of the predictions. We used a combination of edit and Hamming distances, with the latter being more conservative as it only allows substitutions. Interestingly, the Hamming distance contributed most to the sequence similarity in mouse and human, whereas the more flexible edit distance also became important in the fruit fly. This indicates that A-to-I RNA editing is more highly conserved in mouse and human than in fruit fly.

By scanning the whole human genome, it was surprising to find a large number of 41nt-length sequence segments, using 26 228 human editing-sequence contexts as seeds. With a maximum of 3 nucleotide mismatches and no indel, we found 25 487 374 matched segments, revealing that RNA editing may be very frequent in humans. Considering that this number largely exceeds the number of currently known A-to-I RNA editing events and that there might be tissue-specific editing events, it is likely that currently unknown mechanisms control RNA editing. A prediction using only exactly matched sequences yielded 203 202 potential A-to-I RNA editing events. Among those, most were located in noncoding regions. At this point, the role of RNA editing in these regions remains unclear. We also predicted several thousands A-to-I RNA editing events in long non-coding RNA (lncRNA). Further investigations on these specific RNA editing events would be attractive as lncRNA plays an important role in cellular regulation, such as pre-mRNA processing and splicing, transport, translation, and degradation.

As previously reported, A-to-I RNA editing events were rarely found in human pancreatic islets. Nevertheless, we were able to validate 35 A-to-I RNA editing events in this tissue, based on newly analyzed Exome- and RNA- seq data from 12 batches of human islets not included in a previous report from our centre [28]. It is clear that more editing events will be found when the quantity/number of human tissues and samples is increased. This may explain the difference in number of A-to-I RNA editing events reported. We additionally found that the base quality of editing sites in repetitive regions were frequently low or in no-call sites. This is probably due to misalignment of short reads of RNA-seq. Finally, we noticed that several A-to-I events occurred at sites of SNPs. This indicates that RNA editing should be considered as a factor in genome-wide association studies.

In conclusion, we have shown that A-to-I RNA editing events can be predicted by the flanking genome sequences. The very large number of putative editing sites identified in the whole human genome may become a resource, which can be further investigated and validated using targeted deep sequencing or PCR.

## Supporting Information

S1 Dataset Human putative A-to-I RNA editing events. (XLS)

S1 Table The genomic positions of A-to-I RNA editing events used in train sets for fruit fly (DM3). (XLSX)

S2 Table The genomic positions of A-to-I RNA editing events used in train sets for mouse (MM10). (XLSX)

S3 Table The genomic positions of A-to-I RNA editing events used in train sets for human (hg19). (XLSX)

S4 Table Performances of the five-fold cross validation on fruit fly using edit distance and Hamming distance with length of editing-sequence contexts varying from 11-mer to 51-mer. (XLSX)

S5 Table Performances of the binary classifier in the regions of Alu elements and non-Alu elements, respectively. (XLSX)

S6 Table The performance of the prediction on Sanger sequencing validated human RNA editing events. (XLSX)

S7 Table Non-synonymous substitutions in the putative A-to-I RNA editing sites. (XLSX)

S8 Table Validation of 561 putative A-to-I RNA editing events in 184 human islets. (XLSX)

S9 Table Validation of known A-to-I RNA editing events in 184 human islets. (XLSX)
